# Frequency of vaccine‐associated syncope after COVID‐19 vaccination in adolescents

**DOI:** 10.1002/joa3.12721

**Published:** 2022-04-21

**Authors:** Bonpei Takase, Tetsuya Hisada, Nobuyuki Masaki, Masayoshi Nagata, Wataru Shimizu

**Affiliations:** ^1^ Department of Intensive Care Medicine National Defense Medical College Tokorozawa Japan; ^2^ Division of Cardiology Iruma Heart Hospital Iruma Japan; ^3^ Department of Cardiovascular Medicine Nippon Medical School Bunkyo‐ku Japan

**Keywords:** anxiety, side effect, vaccine, viral infection

Coronavirus disease 2019 (COVID‐19) is a global pandemic. Vaccines serve as a key factor in overcoming this serious disease. Since COVID‐19 vaccines are brand new, unexpected side effects are a serious concern, and fear of side effects leads to abstinence from vaccination.

Syncope is defined as the sudden onset of loss of consciousness with collapse and prompt recovery with a possible connection to serious adverse outcomes. Syncope is a common adverse symptom of vaccination.^1^ The prevalence of syncopal episode after COVID‐19 vaccination has been reported,^2^ however, the precise incidence rate and a typical adolescent case with detailed presentation on COVID‐19 vaccine‐induced syncope have not been published to the best of our knowledge. As COVID‐19 vaccination has recently begun in adolescents in Japan, this information would be useful.

We present two typical cases of vasovagal (reflex) syncope in adolescent patients who received the COVID‐19 vaccine manufactured by Pfizer in a consecutive cohort, and we discuss the clinical features and the incidence of syncope caused by vaccination by comparing it with other vaccinations. Written informed consent was obtained from the following patients.

Case 1: A 19‐year‐old healthy man received his first dose of the Pfizer COVID‐19 vaccine, and he fainted 17 min after the vaccine injection without prodrome when he stood up and started to walk after 15 min of waiting at the hall for observation. The patient suddenly lost consciousness and collapsed on the floor. After lying on the floor, he promptly responded to auditory stimuli, and his consciousness returned to normal. His blood pressure was 90/60 mmHg, and his pulse rate was 49 bpm. Intravenous atropine sulfate of 0.5 mg was administered. His consciousness fully recovered, with a normal 12‐lead electrocardiogram. His blood tests, including those examining AST, ALT, CPK, CRP, troponin T, D‐dimer, and complete blood counts, were within normal limits.

Case 2: An 18‐year‐old healthy woman passed out at the hall while waiting for 10 min after the first dose of the Pfizer COVID‐19 vaccine. She suddenly collapsed and lay down on the floor without any prodrome. She was dazed but responded well. Her blood pressure was 84/58 mmHg, and her pulse rate was 58 bpm and regular, with a normal 12‐lead electrocardiogram. Her blood tests and complete blood count results were normal. Intravenous normal saline (500 ml) was rapidly administered and her consciousness fully recovered.

In our institute, 10 421 Pfizer COVID‐19 vaccinations were performed until October 10, 2021. We encountered two patients with syncope that we believe were associated with the vaccination. The calculated prevalence of syncope rate of Pfizer COVID‐19 vaccination was 0.019%, whereas we reviewed the medical records in our institute for the past 5 years and found no syncope or presyncope among 10 246 influenza vaccinations.

Our two patients showed typical conditions of vaccine‐induced syncope which is associated with fear and anxiety caused by vaccine injection,^1^ and reflex syncope is diagnosed. Even if reflex syncope was expected, medical emergency treatments were attempted when the onset of loss of consciousness occurred because of the possible fatal side effects of COVID‐19, although rare, possibly exist. Especially, about the diagnosis of reflex syncope, based on “JCS/JHRS 2022 Guideline on Diagnosis and Risk Assessment of Arrhythmia,” considering differential diagnosis from paroxysmal atrioventricular block is important when syncopal episode has no prodrome observed in our two cases. We will follow our patients to see if they have recurrent syncope.

The adverse event rates of syncope in COVID‐19 in our study are compatible with previous reports from foreign countries.^2^, ^3^ This is more frequent compared with the influenza vaccine. Previously reported side effects of COVID‐19 show that Bell's palsy, Guillain–Barre Syndrome, optic neuritis, idiopathic thrombocytopenic purpura, multiple sclerosis, complex regional pain syndrome, hypercoagulable state, death/sudden unexplained death, acute transverse myelitis, allergic purpura, anaphylaxis/shock, seizure, preterm labor, syncope, and spontaneous abortion are major side effects, while itch, redness, local swelling, local pain, impotence, drowsiness, anorexia, insomnia, diarrhea, nausea, sore throat, headache, joint pain, chills and shiver, fatigue, and muscle pain are minor side effects. According to experiences in the USA,^2^ the clinical characteristics of anxiety‐related adverse events are tachycardia, hyperventilation, dyspnea, chest pain, paresthesia, headache, pallor, or syncope. COVID‐19‐induced adverse events of syncope have been reported to be 0.023%–0.08% worldwide.^2^, ^3^ The total incidence of anxiety‐related syncope after COVID‐19 vaccination has been reported to be 164 times higher than that after the usual influenza vaccination.^4^ There have been several reports on the incidence of immediate adverse events of syncope in a relatively new papillomavirus recombinant vaccine (HPV) (Gardasil®, Merck & Co., Inc.), quadrivalent meningococcal conjugate vaccine (MCV4) (Menactra®, Sanofi Pasteur, Inc.), and acellular pertussis vaccine (Tdap) (Adacel®, Sanofi Pasteur; Boostrix®, GlaxoSmithKline Biologicals), which are relatively new vaccines, much like the COVID‐19 vaccine. The syncopal adverse event rate among these vaccines is 0.0035%–0.078%.^3^, ^5^ These adverse events of syncope are compatible with COVID‐19 vaccination. Another important observation is that adverse events of syncope in COVID‐19 have been reported to be almost twice as frequent in adolescents than in other age groups.^2^, ^3^

Careful observation after COVID‐19 vaccination might be necessary, even after patients leave the vaccine injection sites. Syncope after vaccination did not occur immediately after or during the vaccine injection. It could occur at the end of the 15 min waiting or even after this period, as shown in Case 1. We hope that reporting these two cases will be useful for medical staff at vaccination sites performing COVID‐19 vaccination in adolescents.

Vaccination for coronavirus disease 2019 (COVID‐19) is a significant modality for controlling the worldwide COVID‐19 pandemic. In cases where there are few experiences with these vaccine procedures in adolescents, many concerns have been raised regarding their side effects. We reported two syncope cases out of 10 421 injections of the Pfizer‐manufactured vaccine and discussed the features and frequency of the post‐COVID‐19‐vaccination syncopal episodes by comparing with influenza vaccination. Since syncopal episodes are probably more frequent in COVID‐19 than in usual vaccination and possibly cause injury, the medical staff involved in COVID‐19 vaccination should be aware of the incidence rates and typical features of COVID‐19 vaccine‐induced syncope, especially in adolescents.

## CONFLICT OF INTEREST

The authors declare no COI in this report.

## AUTHOR CONTRIBUTIONS

B. Takase conducted all the steps of this report and manuscript. H. Katsumi and S. Nagata treated these patients and peer reviewed the manuscript, and N. Masaki and W. Shimizu peer reviewed and revised the manuscript.

## ETHICS APPROVAL STATEMENT AND PATIENT CONSENT STATEMENT

Written informed consents were obtained from the patients.

## CLINICAL TRIAL REGISTRATION

Not applicable. The manuscript provides topics on COVID‐19 vaccine and it is written based on the cases we experienced in daily practice so that we obtained written informed consent from the patients but we did not register this manuscript because this is not a clinical study.

## Data Availability

The datasets obtained during the current case report are not publicly available because of the protection of personal information but are available from the corresponding author upon reasonable request.
